# Assessing Metal Exposures in a Community near a Cement Plant in the Northeast U.S.

**DOI:** 10.3390/ijerph120100952

**Published:** 2015-01-19

**Authors:** Zhao Dong, Michael S. Bank, John D. Spengler

**Affiliations:** 1 Department of Environmental Health, Harvard T. H. Chan School of Public Health, 665 Huntington Avenue, Boston, MA 02115, USA; 2 Department of Environmental Conservation, University of Massachusetts, 160 Holdsworth Way, Amherst, MA 01003, USA; E-Mail: mbank@eco.umass.edu; 3 Department of Environmental Health, Harvard T. H. Chan School of Public Health, 401 Park Drive, Landmark Center 406 West, Boston, MA 02215, USA; E-Mail: spengler@hsph.harvard.edu

**Keywords:** cement plant, metal pollution, human exposures, blood, hair, mercury, arsenic, lead, cadmium

## Abstract

Cement production is a major source of metals and metalloids in the environment, while exposures to metals and metalloids may impact human health in the surrounding communities. We recruited 185 participants living in the vicinity of a cement plant in the northeast U.S., and measured the levels of aluminum (Al), arsenic (As), cadmium (Cd), lead (Pb), mercury (Hg), and selenium (Se) in blood and Hg in hair samples from them. A questionnaire was used to assess potential sources of Hg exposure. Multivariate regressions and spatial analyses were performed to evaluate the relative importance of different routes of exposures. The metal concentrations in blood or hair samples of our study participants were comparable to the U.S. general or regional population. Smoking contributed significantly to Cd and Pb exposures, and seafood consumption contributed significantly to Hg and As exposures, while variables related to the cement plant were not significantly associated with metal concentrations. Our results suggest that our study population was not at elevated health risk due to metal exposures, and that the contribution of the cement plant to metal exposures in the surrounding community was minimal.

## 1. Introduction

Many metals or metalloids, such as lead (Pb), mercury (Hg), cadmium (Cd), and Arsenic (As), can lead to toxic effects in human, even at low levels of exposure. For example, many studies have observed neurodevelopmental effects from Hg [1,2] and Pb [3,4] exposures; chronic exposures to Cd in particulate forms have been associated with changes in pulmonary functions [5]; and chronic As exposures in humans may cause skin, lung and bladder cancer [6].

Cement production is one of the key industrial sources of particulate matters (PM) and metals, especially copper (Cu), zinc (Zn), Pb, nickel (Ni), Cd, Hg and As, which are generated from both combustion of fossil fuels and processing of the raw materials [7,8]. It is estimated that cement production accounts for about 9% of global Hg emissions [9]. Most studies on metals from cement plants have focused on ambient environmental levels in the air [10,11] or soil [12,13,14,15], and a few on biomonitoring using plants [16,17]. Very few studies have examined exposures of metals in human near a cement plant [18], and no study has evaluated the contribution to metal exposures through inhalation or ingestion of dust from cement plant in the presence of other primary pathways, such as smoking or diet. Moreover, correlations among different metals, which may reveal potential shared sources, were underexplored. 

**Figure 1:**
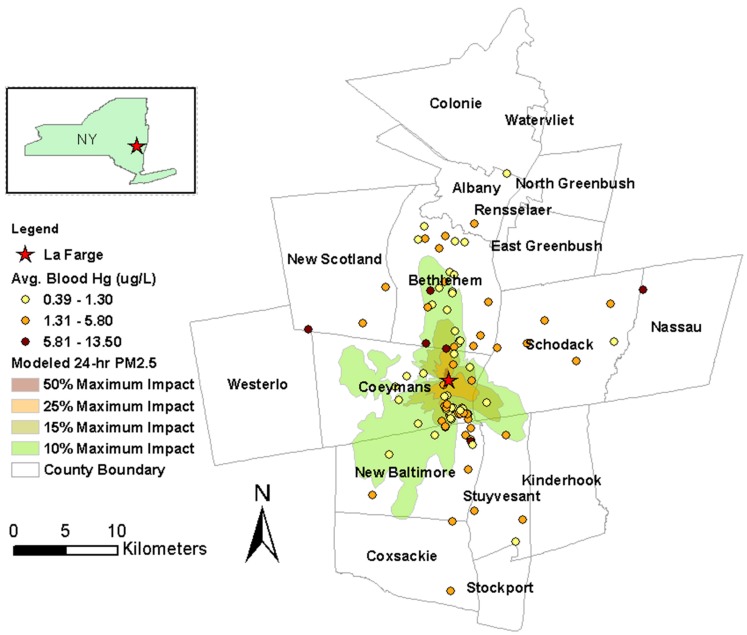
Map of the Ravena study area, showing the location of LaFarge Cement Plant, 24-h PM_2.5_ contours modeled by New York State Department of Health (NYS DOH) [19], and average blood mercury (Hg) in each family.

LaFarge Cement Plant (EPA facility ID: NYD002069557; Figure 1) is located in Ravena, Albany County, New York (NY), surrounded by approximately 15,000 residents and four schools [19]. In the year 2008 alone, the cement plant emitted in total 139 lb of Hg, 160 lb of Pb, 12 lb of Cd, 154 lb of As and 5565 lb of Se into the atmosphere [20]. To address community concerns about possible health impact from metals released by the cement plant, we initiated this study in collaboration with Community Advocates for Safe Emissions (CASE), a local environmental group formed by concerned citizens. The objectives of this study were: to measure specific metals and metalloids in whole blood samples and Hg in hair samples collected from people living in the Ravena community at various distance from the cement plant; to explore potential correlations among these metals; and to evaluate the potential contribution of emissions from the LaFarge cement plant to metal exposures in this community. The overall intent of this investigation was to inform the citizens of Ravena and public health officials of the potential human health risk of this cement plant as well as cement plants in general.

## 2. Experimental Section

### 2.1. Participant Recruitment

We recruited individuals residing within ten miles of Ravena, NY, USA, where the cement plant was located. A total of 185 participants from 120 families, including 17 children (<14 years old), were recruited through email or newspaper advertisement. One blood sample, two duplicate hair samples and phlebotomist data were collected from each participant at a two-day sampling clinic on 15 and 16 May 2010. A total of 172 participants provided blood samples and 153 participants provided hair samples.

All subjects gave their informed consent for inclusion before they participated in the study. The study was conducted in accordance with the Declaration of Helsinki, and the protocol was approved by the Ethics Committee of Harvard T. H. Chan School of Public Health (protocol # CR-18798). 

### 2.2. Mercury Exposure Questionnaire

A mercury exposure questionnaire was developed to assess lifestyle- and work-related exposure of Hg. It was self-administered among participants on the same day as the blood/hair sampling. The questionnaire included questions regarding the participant’s smoking status (current/former/never smoker) and seafood consumption (meals per week), including local wild fish and sushi consumption. It also asked about the presence of industrial dust on the participant’s property (yes/no/uncertain), past mercury spills, potential exposures from work environment, as well as demographic information.

### 2.3. Biomarkers

Total metal concentration in blood was used as a biomarker in this study for assessing exposures of Al, As, Cd, Pb, Hg, and Se. For Cd, Pb and Hg, blood is a commonly used biomarker for exposures up to a few months prior to the collection [21]. For As, urine is generally accepted as the most reliable biomarker to evaluate recent exposures [21], while blood As may better reflect total internal As burden during long-term exposures [22]. 

Hair was also used as a biomarker in our study for evaluating exposures of methylmercury (MeHg), the most bioavailable and toxic form of Hg. Total Hg concentration in hair represents an integration of Hg exposure over a few months, and is considered as a reasonable surrogate for MeHg since 80%–98% of total Hg in hair is present as MeHg [23]. Only the first 2 cm (from scalp) of each hair sample was used for the Hg analysis, which corresponds to Hg exposure two to three months prior to the sample collection.

A report-back letter was sent to each participant in August 2012. It summarized blood metals and hair mercury levels for the entire study population, and provided overall interpretation for these results. According to the NY State Public Health Law, laboratory analyses of human biological samples such as blood or hair must be provided by a licensed physician or otherwise qualified medical worker. Therefore, individual participants obtained their own results through contacting a local New York State Department of Health (NYS DOH) physician, with whom the study team coordinated and communicated about the data.

### 2.4. Metal Analyses

The whole blood metal analyses were conducted by Metametrix Clinical Laboratory, a New York State Clinical Laboratory Evaluation Program (CLEP) certified laboratory, using inductively-coupled plasma mass spectrometry (ICP-MS). For sample preparation, 100 μL of each blood sample was added to 4.9 mL of a diluent, which was deionized water spiked with 0.7% nitric acid and internal standard (*i.e.*, gold, iridium, rhodium and yttrium). Seronorm trace elements whole blood standards (Seronorm L1, L2, and L3) were run approximately every 25 blood samples, and the resulting levels were all within the accepted ranges, while the recoveries were generally within 85%–115%. The detection limit in blood samples was 7.81 μg/L for Al, 2.34 μg/L for As, 0.39 μg/L for Cd, 7.8 μg/L for Hg, 3.1 μg/L for Pb, and 5.0 μg/L for Se. One half of the detection limit concentration was applied to all samples below the detection limit.

Total Hg (THg) levels in hair samples were analyzed by study staff at the Trace Metals Laboratory at the Harvard T. H. Chan School of Public Health, using the method of thermal decomposition, amalgamation and atomic absorption spectrophotometry [24], performed by a DMA-80 Direct Mercury Analyzer (Milestone Inc., Shelton, CT, USA). One method blank and one certified reference material (CRM), GBW-07601 (human hair powder) were tested every 10 samples. Average recovery for the CRM was 114% with an RSD of 7.5%. The detection limit for hair Hg was 0.5 ng/g and all hair samples were above this level.

### 2.5. Data Analyses

Each participant was geocoded based on home address. A spatial join was performed on the data with 24-h PM_2.5_ (particulate matters with a diameter of 2.5 μm or less) contours modeled by NYS DOH [19], in order to determine whether a participant was located within or outside of a contour zone. These contours were generated by an air dispersion model (AERMOD, developed by the U.S. EPA) and included maximum (worst-case) levels of PM_2.5_ emitted into the air from the cement plant (under current plant operation) at ground level. The distance (in km) of each participant’s residence to the cement plant was calculated, and the direction quadrant (NE, NW, SE or SW) of each participant in relation to the plant was also determined. All spatial analyses were conducted in ArcGIS 10.1.

Blood metals and hair Hg were natural log-transformed because the distributions were positively-skewed. A Spearman’s correlation coefficient was calculated for each pair of blood metals. Using each of the seven metal endpoints (blood Al, As, Cd, Pb, Hg, Se, and hair Hg) as the outcome variable, a multivariate linear regression was performed, including age, gender, education, body mass index (BMI), smoking status, dust on property, seafood consumption, distance to the plant, quadrant, 24-h PM_2.5_ contour as covariates (Table 1). The interactions between quadrant and distance and between 24-hour PM_2.5_ contour and distance were also tested. Since many participants were from the same family, a linear mixed model was also considered designating family as the random intercept. The results from this model led to similar conclusions but were less robust due to much fewer degrees of freedom, so only the results from the regular model were reported. RStudio 0.98.978 (with R 3.1.1) was used to perform all statistical analyses.

**Table 1:** Hair Hg levels (μg/g) by age, gender, education, body mass index (BMI), smoking status, dust on property, seafood consumption, distance to La Farge, quadrant, 24-h PM_2.5_ contour, and housing density.

Variables	N	Mean ± SD	Range	Percentiles					
				25th	50th	75th
**All**	153	0.44 ± 0.46	0.019–2.6	0.16	0.33	0.53
**Age ^a^ (years)**						
Q1 ( 3–35)	37	0.25 ± 0.25	0.019–1.1	0.060	0.17	0.33
Q2 (35–51)	41	0.34 ± 0.28	0.035–1.1	0.14	0.26	0.46
Q3 (51–60)	38	0.60 ± 0.50	0.023–2.2	0.25	0.48	0.71
Q4 (60–86)	37	0.58 ± 0.61	0.051–2.6	0.27	0.40	0.57
**Gender**						
Male	73	0.48 ± 0.48	0.028–2.6	0.21	0.35	0.57
Female	80	0.40 ± 0.43	0.019–2.4	0.14	0.27	0.50
**Education**						
High school or less	31	0.38 ± 0.42	0.039–2.2	0.16	0.28	0.48
Some college	28	0.45 ± 0.47	0.019–2.4	0.17	0.35	0.58
College graduate	54	0.48 ± 0.52	0.035–2.6	0.21	0.33	0.53
Advanced degree	31	0.48 ± 0.40	0.051–2.0	0.18	0.40	0.65
**BMI (kg/m^2^)**						
11–25	63	0.43 ± 0.46	0.019–2.4	0.098	0.32	0.57
25–30	49	0.52 ± 0.52	0.028–2.6	0.22	0.36	0.57
30–55	41	0.37 ± 0.35	0.023–2.2	0.14	0.33	0.47
**Smoking Status**						
Current smoker	11	0.33 ± 0.21	0.071–0.77	0.20	0.26	0.43
Former smoker	50	0.61 ± 0.59	0.035–2.6	0.26	0.42	0.71
Never smoker	92	0.36 ± 0.36	0.019–2.2	0.10	0.26	0.49
**Dust on Property**						
Yes	57	0.36 ± 0.38	0.023–2.2	0.12	0.23	0.47
No	34	0.55 ± 0.53	0.050–2.4	0.23	0.40	0.66
Unsure	44	0.47 ± 0.50	0.035–2.6	0.14	0.36	0.60
**Seafood Consumption (per week)**						
0–1 meal	45	0.23 ± 0.19	0.019–0.84	0.071	0.19	0.34
1–2 meals	65	0.48 ± 0.44	0.028–2.2	0.22	0.36	0.57
2–9 meals	30	0.75 ± 0.64	0.055–2.6	0.30	0.51	0.91
**Distance to La Farge ^a^ (km)**						
Q1 (0.71–3.1)	37	0.40 ± 0.44	0.040–2.2	0.16	0.23	0.47
Q2 (3.1–4.3)	32	0.41 ± 0.39	0.023–2.2	0.24	0.33	0.48
Q3 (4.3–8.9)	38	0.49 ± 0.55	0.019–2.6	0.077	0.23	0.66
Q4 (8.9–99)	36	0.45 ± 0.44	0.039–2.4	0.29	0.42	0.57
**Quadrant (relative to La Farge)**						
Northwest	30	0.61 ± 0.60	0.039–2.4	0.24	0.42	0.76
Northeast	44	0.31 ± 0.29	0.019–1.5	0.097	0.24	0.41
Southwest	24	0.39 ± 0.43	0.049–2.2	0.20	0.27	0.48
Southeast	45	0.48 ± 0.46	0.023–2.6	0.20	0.36	0.53
**Modeled 24-h PM_2.5_ contour**						
Out of contour	53	0.38 ± 0.50	0.039–2.6	0.23	0.38	0.57
Within 10% maximum impact	50	0.33 ± 0.46	0.019–2.2	0.13	0.33	0.53
Within 15% maximum impact	37	0.23 ± 0.40	0.047–2.2	0.16	0.23	0.40
Within 25% maximum impact	3.00	0.28 ± 0.26	0.092–0.61	0.18	0.28	0.44

**^a^** Q1–Q4 refer to first through fourth quartile ranges.

## 3. Results

### 3.1. Participant Characteristics

Our population was evenly distributed in age and gender, with a median age of 51 and 55% female, higher than the median age of 39 and comparable to 52% female in Albany County [25], NY, USA. Most (77%) participants had at least some college education, higher than the average of 66% in Albany County. About 30% of the participants were overweight and 27% were obese, with a median BMI of 26 kg/m^2^. The majority (92%) of our population were not active smokers, within which 59% participants never smoked and 33% were former smokers.

About 42% participants confirmed that industrial dust had landed on their properties, while 33% were uncertain. The distance from the cement plant to participants’ home ranged from 0.7 to 9.9 km, with a median of 4.2 km. An approximately equal number of participants lived in the north (51%) and south of the plant, while more participants (64%) lived to the east of the plant.

Seafood consumption of our participants ranged from 0 to 9 meals per week, with a median of one meal per week, which is lower than EPA’s recommended consumption level of two or three servings per week of fish with low contaminant levels for pregnant or breastfeeding women [26]. Very few of our participants consumed local wild fish or sushi over the previous six months. No recent Hg spills or work-related Hg exposures were reported by the participants.

### 3.2. Metals in Blood

For Cd and Pb, the levels in whole blood samples from our study population were below or comparable to the general population in the USA (Table 2). For Hg in blood, levels found in our population were higher than the national data [21], but the levels in female participants were comparable to the levels seen in women located in the northeastern region of the USA [27]. For Hg in hair, national data were only available for women [28], to which levels in our female participants were comparable. There were no national data available for Al, As and Se in blood, but previous literature suggests a reference range of 2–8 μg/L for blood Al [29] and 0.08–0.25 mg/L for blood Se [30]. For As, the blood levels in unexposed individuals is usually less than 1 μg/L [31], while levels up to 12 μg/L can be considered as normal [32]. Overall, the metals found in blood or hair samples of our study population were within the typical ranges of the general or regional population in the USA.

Thirteen participants had Hg levels slightly above the NYS DOH Heavy Metals Registry reportable level of 5 μg/L, and they were informed of their individual results through the Registry. A total of 11 participants (including five women and no children) had blood Hg levels above 5.8 μg/L, and 10 participants (including five women and no children) had hair Hg levels above 1.1 μg/g, which correspond to U.S. EPA’s reference dose (RfD) of 0.1 μg/kg/d MeHg for women of child-bearing age and children [33]. Five people (including three males) had a hair Hg value above 2 μg/g, a level associated with cardiovascular effects in middle-aged men in some studies [34,35]. 

Two participants had blood Pb levels above the NYS DOH reportable level of 10 μg/dL at the time of the study, and NYS DOH was notified regarding these findings. Five participants, including no women or children, had a blood Pb level above 5 μg/dL, which is the CDC (U.S. Centers for Disease Control) reference level for blood Pb in children. No participant had a blood Cd level above the NYS DOH reportable level of 10 ng/mL.

### 3.3. Correlation among Metals

There were highly significant correlations between Pb and Cd, and between As and Hg in blood samples of our population (Table 3). In addition, significant correlations were found between As and Pb, As and Se, and between Al and Cd. There were also marginally significant correlations between Al and Cd, and between Hg and Se. All significant correlations were positive.

**Table 2:** Levels of Al, As, Cd, Pb, Hg and Se in blood and Hg in hair, compared to NHANES national data.

Metal		N	Geometric Mean (95% CI)	Percentiles			
				50th	75th	90th	95th
Blood Al (μg/L)							
	Ravena, NY	172	4.6 (3.8–5.4)	4.3	5.8	15	17
Blood As (μg/L)							
	Ravena, NY	172	2.2 (2.0–2.4)	2.5	3.4	5.1	6.5
Blood Cd (μg/L)							
	Ravena, NY	172	0.30 (0.27–0.32)	0.20	0.50	0.80	0.94
	National data **^a^**	8372	0.30 (0.29–0.32)	0.30	0.50	1.1	1.6
Blood Pb (μg/dL)							
	Ravena, NY	172	1.1 (0.96–1.2)	1.2	1.7	2.7	3.5
	National data **^a^**	8373	1.4 ( 1.4–1.5)	1.5	2.1	3.2	4.2
Blood Hg (μg/L)							
	Ravena, NY	172	1.3 (1.1–1.4)	1.3	2.5	4.0	6.2
	National data **^a^**	8,373	0.80 (0.70–0.90)	0.80	1.7	3.3	4.9
	Ravena, women only	96	1.2 (0.96–1.4)	1.2	2.4	4.1	7.1
	National, women only **^a^**	4,241	0.78 (0.69–0.89)	0.80	1.6	3.0	4.4
	NHANES women, northeast USA **^b^**	820	1.1 (0.84–1.6)	1.1	2.6	5.2	8.2
Hair Hg (μg/g)							
	Ravena, NY	153	0.28 (0.24–0.33)	0.33	0.53	0.86	1.3
	Ravena, women only	80	0.25 (0.20–0.31)	0.27	0.50	0.75	1.2
	NHANES women **^c^**	1,726	0.20 (0.16–0.24)	0.19	0.42	1.1	1.7
Blood Se (mg/L)							
	Ravena, NY	172	0.17 (0.16–0.17)	0.17	0.18	0.21	0.22

**^a^** Reference [21]; **^b^** Reference [27]; **^c^** Reference [28].

**Table 3:** Correlation matrix among Al, As, Cd, Pb, Hg, and Se in blood.

Metals	Al	As	Cd	Pb	Hg	Se
**Al**	1					
**As**	0.075	1				
**Cd**	0.18 *	0.14 **^**	1			
**Pb**	−0.059	0.22 **	0.34 ****	1		
**Hg**	−0.040	0.53 ****	0.024	0.31	1	
**Se**	−0.018	0.21 **	−0.041	0.090	0.14 **^**	1

**^**: *p* < 0.1; *: *p* < 0.05; **: *p* < 0.01; ****: *p* < 0.0001.

### 3.4. Multivariate Linear Regression

The results from the regression differed among metals (Table 4). After accounting for all other variables, age was a significant predictor of blood Cd, Pb, Hg and hair Hg, and a ten-year increase in age was associated with an increase of 8.7% (calculated as (e^model estimate^ − 1) × 100, the same below) in blood Cd, 31% in blood Pb, 28% in blood Hg and 27% in hair Hg, respectively. Gender was significantly associated with blood Pb only, of which the levels in men exceeded women by 36%. BMI was a significant predictor for Pb, Hg and Se in blood, and a 1 kg/m^2^ increase in BMI was associated with a decrease of 4.0% in blood Pb, 2.8% in blood Hg and 0.66% in blood Se. An association with level of education was observed with blood Se, but only within people with some college, who had 8%–13% higher blood Se than the other groups. Smoking was a significant predictor for blood Cd and Pb: compared to never smokers, current smokers had 230% higher blood Cd and 120% higher blood Pb. Seafood consumption was significantly associated with As, Cd, Hg in blood and Hg in hair; consuming one additional meal per week corresponds to 21% higher blood As, 10% lower blood Cd, 26% higher blood Hg and 21% higher hair Hg. Quadrant was significantly associated with blood Hg only, and people living southeast or southwest of the cement plant had 77% or 110% lower blood Hg than people living northwest of the plant. Dust on property, distance to the plant, and PM_2.5_ contour were not significant predictors for any of the metals. 

The quadrant × distance interaction was not significant for any of the metals (results not shown in Table 4). The PM_2.5_ contour × distance interaction was significant for blood Hg only, and the estimates were negative and decreased from −0.063 to −1.97 from the 10% to 25% maximum impact contours. 

The adjusted R-squared (adj-R^2^) of the regression model was 0.039 for blood Al, indicating that none of the considered variables helped to explain the variability observed in the data. For the other metals, the model goodness-of-fit was best for blood Pb (adj-R^2^ = 0.41) and blood Hg (0.37), followed by blood Cd (0.27), hair Hg (0.21), blood As (0.15), and blood Se (0.13).

## 4. Discussion

In this study, we evaluated exposures to a variety of metals in participants residing near LaFarge Cement Plant in Ravena, NY, USA and assessed the contribution to metal exposures from different sources. We found that exposures of our participants to toxic metals such as Pb, Cd and Hg were within the range expected for the general U.S. population or for the regional population. Overall, it is unlikely that local residents living near the cement plant had elevated health risks from metal exposures.

We also found that the cement plant, even though emitting a substantial amount of metals to the atmosphere, had little contribution to metal exposures examined in this study, after accounting for the effects from smoking and seafood consumption. Dust on property, distance and direction quadrants to the plant, as well as modeled 24-h PM_2.5_ contours, were not significantly associated with our metal biomarkers, except for Hg in blood, for which participants living in the northwest of the cement plant had higher levels than the other participants. However, this quadrant effect was observed only for Hg in blood but not for hair Hg, and was likely caused by just a few people with high Hg levels (Figure 1).

**Table 4:** Multivariate regression on natural log-transformed blood Al, As, Cd, Pb, Hg, Se and Hair Hg. Numbers represent model estimates, with 95% confidence intervals included in parentheses.

Variables	Blood Al (μg/L)	Blood As (μg/L)	Blood Cd (μg/L)	Blood Pb (μg/L)	Blood Hg (μg/L)	Hair Hg (μg/L)	Blood Se (μg/L)
	(n = 109, adj-R^2^ = 0.039)	(n = 109, adj-R^2^ = 0.15)	(n = 109, adj-R^2^ = 0.27)	(n = 109, adj-R^2^ = 0.41)	(n = 109, adj-R^2^ = 0.37)	(n = 94, adj-R^2^ = 0.20)	(n = 108, adj-R^2^ = 0.13)
**Age (years)**	−0.0015 (−0.0098, 0.0068)	0.0060 (−0.0017, 0.014)	0.0083 (0.0021, 0.015) **	0.027 (0.019, 0.035) ***	0.025 (0.016, 0.035) ***	0.024 (0.011, 0.036) ***	0.0010 (−0.00084, 0.0029)
**Gender (Referent = Female)**							
Male	−0.12 (−0.36, 0.12)	−0.13 (−0.35, 0.097)	−0.16 (−0.34, 0.025) ^	0.31 (0.078, 0.54) **	−0.023 (−0.30, 0.25)	0.22 (−0.13, 0.57)	−0.033 (−0.087, 0.022)
**Education (Referent = High school or less)**							
Some college	0.22 (−0.21, 0.66)	0.30 (−0.11, 0.70)	0.15 (−0.17, 0.48)	0.33 (−0.085, 0.75)	0.079 (−0.42, 0.58)	0.0051 (−0.65, 0.67)	0.12 (0.018, 0.21)
College graduate	0.088 (−0.27, 0.45)	0.25 (−0.081, 0.59)	−0.089 (−0.36, 0.18)	0.12 (−0.23, 0.46)	−0.20 (−0.61, 0.21)	−0.29 (−0.85, 0.27)	0.032 (−0.049, 0.11)
Advanced degree	0.078 (−0.33, 0.49)	0.33 (−0.051, 0.71) ^	0.051 (−0.25, 0.36)	0.17 (−0.22, 0.56)	−0.26 (−0.73, 0.21)	−0.32 (−0.97, 0.33)	−0.013 (−0.11, 0.080) *
BMI (kg/m^2^)	0.017 (−0.0051, 0.040)	−0.0010 (−0.022, 0.020)	−0.011 (−0.027, 0.0062)	−0.039 (−0.061, −0.018) ***	−0.028 (−0.054, −0.0020) *	−0.013 (−0.048, 0.022)	−0.0066 (−0.012, −0.0015) *
**Smoking Status (Referent = Never smoker)**							
Current smoker	−0.21 (−0.74, 0.32)	−0.29 (−0.79, 0.20)	1.2 (0.79, 1.6) ***	0.79 (0.28, 1.3) **	−0.44 (−1.1, 0.17)	0.25 (−0.56, 1.1)	−0.035 (−0.16, 0.086)
Former smoker	−0.21 (−0.47, 0.045)	−0.058 (−0.3, 0.18)	0.085 (−0.11, 0.28)	0.085 (−0.16, 0.33)	0.26 (−0.038, 0.55) ^	0.37 (−0.018, 0.76) ^	0.016 (−0.042, 0.075)
Seafood Consumption (meals/week)	0.061 (−0.046, 0.17)	0.19 (0.094, 0.29) ***	−0.095 −0.17, −0.015) *	0.023 (−0.079, 0.13)	0.23 (0.11, 0.35) ***	0.19 (0.039, 0.35) *	0.012 (−0.012, 0.036)
**Dust on Property (Referent = No)**							
Yes	−0.023 (−0.41, 0.36)	−0.032 (−0.39, 0.33)	−0.19 (−0.48, 0.10)	−0.11 (−0.48, 0.26)	0.0040 (−0.44, 0.45)	−0.50 (−1.1, 0.12)	0.011 (−0.076, 0.099)
Unsure	−0.26 (−0.59, 0.074)	−0.00027 (−0.31, 0.31)	−0.12 (−0.37, 0.12)	−0.12 (−0.43, 0.20)	0.092 (−0.29, 0.47)	−0.30 (−0.81, 0.22)	−0.0031 (−0.078, 0.072)
Distance to La Farge (km)	0.00022 (−0.015, 0.015)	0.0011 (−0.013, 0.015)	0.0020 (−0.0091, 0.013)	−0.0051 (−0.019, 0.0091)	0.0060 (−0.011, 0.023)	−0.013 (−0.071, 0.044)	−0.00029 (−0.0036, 0.0031)
**Quadrant Relative to La Farge (Referent = Northwest)**							
Northeast	−0.15 (−0.52, 0.22)	−0.14 (−0.48, 0.21)	−0.13 (−0.40, 0.15)	0.0054 (−0.35, 0.36)	−0.42 (−0.85, −0.00044) ^	−0.31 (−0.86, 0.25)	−0.099 (−0.18, −0.016) *
Southwest	−0.37 −0.76, 0.025) ^	−0.22 (−0.58, 0.15)	0.090 (−0.20, 0.38)	0.17 (−0.20, 0.54)	−0.57 (−1.0, −0.12) *	−0.091 (−0.69, 0.51)	−0.016 (−0.10, 0.073)
Southeast	−0.27 (−0.70, 0.17)	−0.23 (−0.63, 0.18)	−0.066 (−0.39, 0.26)	−0.11 (−0.53, 0.31)	−0.73 (−1.2, −0.23) **	−0.41 (−1.1, 0.29)	−0.073 (−0.17, 0.026)
**Modeled 24-h PM_2.5_ Contour (Referent = Out of contour)**							
Within 10% maximum impact	−0.20 (−0.55, 0.14)	−0.016 (−0.34, 0.30)	−0.090 (−0.35, 0.17)	−0.24 (−0.57, 0.091)	0.20 (−0.020, 0.59)	0.14 (−0.41, 0.68)	0.0069 (−0.071, 0.085)
Within 15% maximum impact	−0.21 (−0.68, 0.25)	0.055 (−0.38, 0.49)	−0.10 (−0.45, 0.24)	−0.099 (−0.54, 0.35)	0.20 (−0.34, 0.73)	0.056 (−0.70, 0.81)	0.012 (−0.093, 0.12)
Within 25% maximum impact	−0.30 (−1.1, 0.53)	0.010 (−0.76, 0.79)	0.28 (−0.34, 0.90)	−0.43 (−1.2, 0.37)	0.65 (−0.30, 1.6)	0.40 (−0.98, 1.8)	0.052 (−0.14, 0.24)

**^**: *p* < 0.1; *: *p* < 0.05; **: *p* < 0.01; *** *p* < 0.001.

The same regression model on blood Hg excluding two participants with the top 1% of blood Hg levels (≥10.5 μg/L) generated similar results to the original model in Table 1, while this quadrant effect was no longer significant. In addition, the primary wind comes from the south and northeast in this area [20], and participants living in the northwest quadrant were actually less likely to report presence of dust on their properties compared to the other participants according to our data. Meanwhile, the negative interaction between PM_2.5_ contour and distance for blood Hg suggests that for participants living closer to the cement plant, the PM_2.5_ contours had a larger association with their blood Hg levels and this effect increased from the outer to inner contours, even though being within the contours was not associated significantly with higher blood Hg levels. This indicates that the cement plant emissions have the potential to impact exposures, but in this case the overall risk is low. In general, the four covariates (dust, distance, quadrant, PM_2.5_ contours) included in our regression models that might be related to the cement plant together explained less than 3% of variability (calculated as the change in adj-R^2^ with or without the variables) observed in the metal concentrations.

According to our regression analysis, contributions from different sources varied among metals. For Hg, seafood consumption was an important source, explaining 7.5% of variability in blood Hg and 2.8% in hair Hg. This is consistent with the observation in the general U.S. population, in whom seafood consumption is the most important non-occupational source of MeHg [28]. Seafood consumption was also found to contribute significantly to As exposure in this study, explaining 9.5% of variability in blood As, which was expected since blood As generally contains more organic As that tends to bioaccumulate in fish [36]. For Cd and Pb, smoking was a more significant contributor to blood levels, explaining 21% and 3.9% of the observed variability, respectively. This is supported by numerous literatures that found significant amount of Cd and Pb in tobacco smoke [37,38,39].

The positive correlations we found among metals supported the potential common sources mentioned above. The strongest correlations were found between Hg and As, and between Cd and Pb, and this may be related to shared pathways of seafood consumption and smoking. The less strong but also significant correlations between As and Pb may suggest exposure to lead arsenate pesticides. The co-occurrence of As and Pb has been commonly found in U.S. soils due to wide-spread use of these pesticides in orchards since the 1900s [40,41]. In addition, the significant correlation between As and Se may reflect shared dietary sources for these two metals, including the consumption of fish, meat or cereals [42].

Unlike other metals, Al is quite ubiquitous in the environment [43,44], thus it is not surprising that none of the covariates tested in our regression model for blood Al was significant. Since Al tends to co-vary with other metals, it is often used as a reference metal for the background levels. Using the typical Al concentration in human whole blood (2–8 ug/L) and blood concentrations of Hg, Pb and Cd in the general U.S. population, we can calculate the enrichment factor (EF), used commonly as an indicator for metal pollution relative to background levels, for each metal (Me) in blood samples of our study participants through the following equation [45]:

(1) $$\text{EF}=\frac{{\left(\text{Me}/\text{Al}\right)}_{\text{our study}}}{{\left(\text{Me}/\text{Al}\right)}_{\text{background}}}$$

The average EF is 0.7–2.8 for Hg, 0.3–1.4 for Pb and 0.4–1.7 for Cd, suggesting very little enrichment (EF < 3) of these metals in our participants relative to the general population.

According to our results, some biological factors, such as age and BMI, may also have played a role in metal exposures in our participants. The significant increase in in blood Cd, Pb, Hg and hair Hg levels over age might be caused by a change in lifestyle or seafood consumption as a function of age [46], or by long-term accumulation of metals in human body during chronic exposures [47]. The negative effect of BMI on blood Pb, blood Hg and hair Hg concentrations might be related to increasing blood volume over body weight [48]. 

This study was constrained by its small sample size and non-representative sampling regime. Our study participants mostly consisted of volunteers who were concerned about the health of themselves or their children, or about the health risks of living in this community in general, thus generalization to the full population is limited. However, this selection bias would likely lead to an over-estimation of the exposures, while the levels observed in this study were comparable to those found in the general population. Therefore, we might still be able to draw the conclusion that this community is not at elevated health risks from metal exposures. 

Our exposure questionnaire only asked about frequency of overall seafood consumption, while including very limited species-specific information and no portion size. This could have led to uncertainties in our exposure assessment. For example, even though the average number of seafood meals of our participants did not exceed those of the U.S. general population, our participants may have consumed larger portions, and/or eaten species of higher Hg levels, contributing to their slightly higher blood Hg levels compared to the U.S. general population. 

The typical exposure levels and low impact from the cement plant found in this study were consistent with previous studies assessing health risks in populations living in the vicinity of a cement plant [11,49,50] and the public health assessment conducted by NYS DOH [19]. It should be noted that the cement plant-related variables we tested in this study only represent inhalation, ingestion or dermal exposures to metals emitted in the gaseous form or associated with particulate matter, which may not be the most relevant pathway of concern. For instance, MeHg is the most important Hg species regarding human exposure and the predominant species represented by the blood or hair biomarkers used in this study, while the majority of Hg emission from cement plants is in the inorganic form [51]. Other routes of Hg exposure may exist, including exposures to soil, water or seafood contaminated by cement plant emissions. However, they should be relatively unimportant in this community, since environmental samples (including soil, water and biota tissues) collected at Ravena did not show elevated metal concentrations compared to typical background levels [20], and according to our questionnaire, consumption of locally-caught wild fish in our participants was rare.

Based on the results from this study, there is lack of evidence for the cement plant being a significant source of metal exposures to this local community. Nevertheless, the legacy of high emissions has certainly contributed to atmospheric deposition of Hg and other metals in the northeastern USA, while fugitive and stack emissions of alkaline cement dust and PM_2.5_ contributed to local deposition and elevated airborne particulate concentrations. The rebuilding of the Ravena cement plant starting in April 2014, with plant modernization and improved emission controls as part of a 2010 settlement with state and federal officials, will improve local air quality while greatly reducing the discharge of Hg to no more than 59 lb a year by 2017, a 58% cut from the 2008 emission [52].

## 5. Conclusions

This study explored the potential human health impact of the Ravena cement plant on its nearby community. Despite its high metal emissions to the atmospheric environment, the cement plant had relatively small contribution to human metal exposures compared to other sources such as seafood consumption and smoking, and evaluation of the biomarkers suggests that our study participants were not at elevated health risk associated with metal exposures.
